# Supplementary material for: An open-source framework for large-scale, flexible evaluation of biomedical text mining systems
Source: J Biomed Discov Collab. 2008 Jan 29;3:1. doi: 10.1186/1747-5333-3-1 (PMC2276192; doi:10.1186/1747-5333-3-1)
Supplement: Additional file 1 — Evaluation platform source code. The additional file contains the software evaluation framework discussed in this paper. The most current release can be downloaded from SourceForge.net [1]. See the accompanying README file for instructions on installation and use. Also included in the distribution is a collection of UIMA wrappers for some commonly used BioNLP tools and annotated corpora. [file 1747-5333-3-1-S1.zip › bionlp_uima_repository-1.0.1/docs/api/edu/uchsc/ccp/util/nlp/parser/corpus/TexasCorpusParser.html]

TexasCorpusParser (Center for Computational Pharmacology Util/UIMA API)


|  |  |  |
| --- | --- | --- |
| **Overview** \| **Package** \| **Class** \| **Tree** \| **Deprecated** \| **Index** \| **Help** | |  |
| **PREV CLASS**   **NEXT CLASS** | **FRAMES**    **NO FRAMES**     **All Classes** |  |
| SUMMARY: NESTED \| FIELD \| CONSTR \| METHOD | DETAIL: FIELD \| CONSTR \| METHOD |  |


---


## edu.uchsc.ccp.util.nlp.parser.corpus Class TexasCorpusParser

```
java.lang.Object
  edu.uchsc.ccp.util.nlp.parser.corpus.CorpusParser
      edu.uchsc.ccp.util.nlp.parser.corpus.TexasCorpusParser
```

**All Implemented Interfaces:**: Iterator

---

``` public class TexasCorpusParser extends CorpusParser ```

A parser for the Texas corpus. This class outputs the original text and accompanying annotations for each Texas corpus document. Titles and
abstracts are outputted as separate documents.  
  
Output Classes:  

- protein

**Author:**
:   williamb

---

| **Field Summary** | |
| --- | --- |
| `static int` | `DOCUMENT_COLLECTION_ID` |


**Constructor Summary**

`TexasCorpusParser(String corpusDirectory)`
  
          Initialize a new parser for the texas corpus.


| **Method Summary** | |
| --- | --- |
| `void` | `close()` |
| `static List<String[]>` | `getDocuments()` |
| `static List<TextAnnotation>` | `getTextAnnotations()` |
| `boolean` | `hasNext()` |
| `static void` | `main(String[] args)`             args[0] = texas corpus directory |
| `GenericDocument` | `next()` |
| `static void` | `parseTexasCorpusDirectory(String directory)`             Parse the corpus and store all annotations and documents into the annotation database |
| `static void` | `parseTexasCorpusFile(String documentID, BufferedReader br)`             This method parses the Texas corpus. |

**Methods inherited from class edu.uchsc.ccp.util.nlp.parser.corpus.CorpusParser**

`createTextAnnotation, error, remove, warn`

**Methods inherited from class java.lang.Object**

`clone, equals, finalize, getClass, hashCode, notify, notifyAll, toString, wait, wait, wait`

**Field Detail**

### DOCUMENT\_COLLECTION\_ID

```
public static final int DOCUMENT_COLLECTION_ID
```

**See Also:**: Constant Field Values


**Constructor Detail**

### TexasCorpusParser

```
public TexasCorpusParser(String corpusDirectory)
```

:   Initialize a new parser for the texas corpus.

    **Parameters:**: `corpusDirectory` -


**Method Detail**

### hasNext

```
public boolean hasNext()
```

:   **Specified by:**: `hasNext` in interface `Iterator` **Specified by:**: `hasNext` in class `CorpusParser`

---


### next

```
public GenericDocument next()
```

:   **Specified by:**: `next` in interface `Iterator` **Specified by:**: `next` in class `CorpusParser`

---


### getTextAnnotations

```
public static List<TextAnnotation> getTextAnnotations()
```

---


### getDocuments

```
public static List<String[]> getDocuments()
```

---


### parseTexasCorpusDirectory

```
public static void parseTexasCorpusDirectory(String directory)
                                      throws IOException
```

:   Parse the corpus and store all annotations and documents into the annotation database

    :   **Parameters:**: `directory` - **Throws:**: `IOException`

---


### parseTexasCorpusFile

```
public static void parseTexasCorpusFile(String documentID,
                                        BufferedReader br)
                                 throws IOException
```

:   This method parses the Texas corpus. TODO: This should be replaced at some point with the Iterator methods

    :   **Parameters:**: `documentID` -: `br` - **Throws:**: `IOException`

---


### close

```
public void close()
           throws IOException
```

:   **Specified by:**: `close` in class `CorpusParser`
:   **Throws:**: `IOException`

---


### main

```
public static final void main(String[] args)
```

:   args[0] = texas corpus directory

    :   **Parameters:**: `args` -


---


|  |  |  |
| --- | --- | --- |
| **Overview** \| **Package** \| **Class** \| **Tree** \| **Deprecated** \| **Index** \| **Help** | |  |
| **PREV CLASS**   **NEXT CLASS** | **FRAMES**    **NO FRAMES**     **All Classes** |  |
| SUMMARY: NESTED \| FIELD \| CONSTR \| METHOD | DETAIL: FIELD \| CONSTR \| METHOD |  |


---
